# Supplementary material for: A new patient‐derived iPSC model for dystroglycanopathies validates a compound that increases glycosylation of α‐dystroglycan
Source: EMBO Rep. 2019 Sep 30;20(11):e47967. doi: 10.15252/embr.201947967 (PMC6832011; doi:10.15252/embr.201947967)
Supplement: Supplementary file 1 — Appendix [file EMBR-20-e47967-s001.pdf]

Appendix

Table of Contents

Appendix Figure S1. Gene expression analysis of pluripotency-associated genes in FKRPA455D-iPSC lines by qPCR. 3

Appendix Figure S2. PCR genotyping of iPSC clones after positive/negative selection. 4

Appendix Figure S3. Knocking in FKRP c.1364C>A (p.A455D) mutation by CRISPR/Cas9 in WT-iPSCs to generate isogenic FKRP mutated-iPSCs. 5

Appendix Figure S4. Characterization of the isogenic pair of WT- and FKRP mutated-iPSCs 7

Appendix Figure S5. Uncropped images of representative immunoblots. 8

Appendix Figure S6. Gene expression analysis in FKRP- and corrected-NSCs treated with 4BPPNit. ... 9

Appendix Table S1. *FKRP* on-target locus and top 5 predicted off-target sites. 10

Appendix Table S2. Targeted gene correction by CRISPR/Cas9. 10

Appendix Table S3. Targeted gene mutation by CRISPR/Cas9. 11

Appendix Table S4. Primers used for constructing targeting donor vector by Gibson Assembly and generating DNA fragments containing sgRNA target sequence. 12

Appendix Table S5. Primers used for PCR genotyping 13

Appendix Table S6. Primers used for qPCR. 13

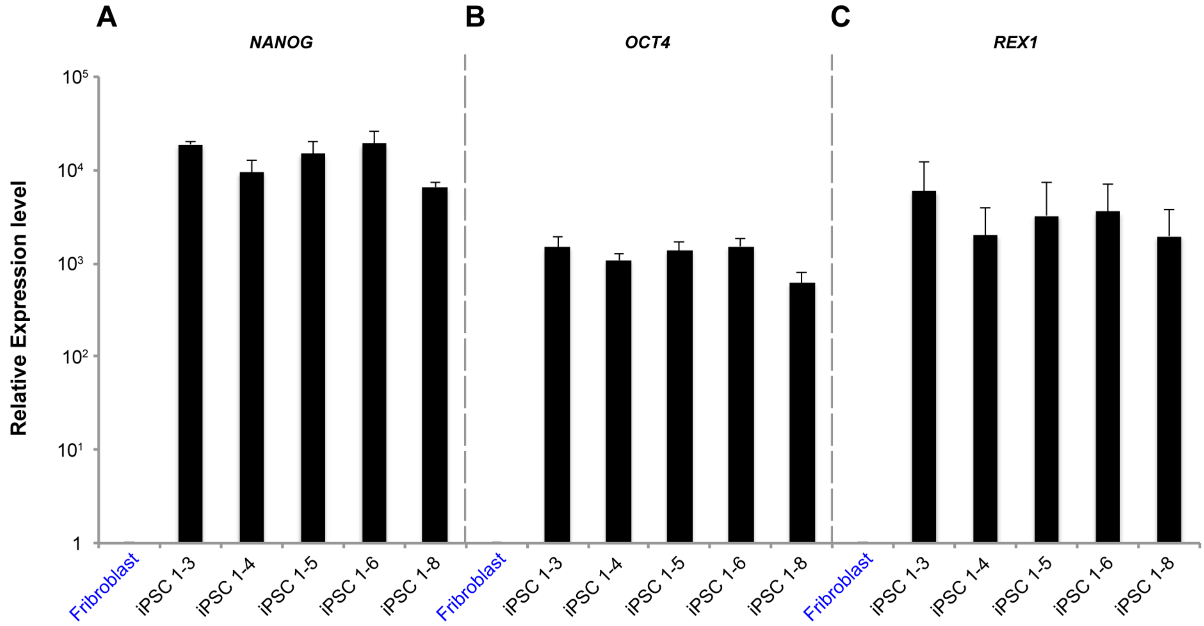

Appendix Figure S1. Gene expression analysis of pluripotency-associated genes in FKRPA455D-iPSC lines by qPCR. Endogenous expression levels of *NANOG* (A), *OCT4* (B) and *REX1* (C) in the $FKRP^{A455D}$-iPSC lines are relative to the parental fibroblast line from which they were derived.

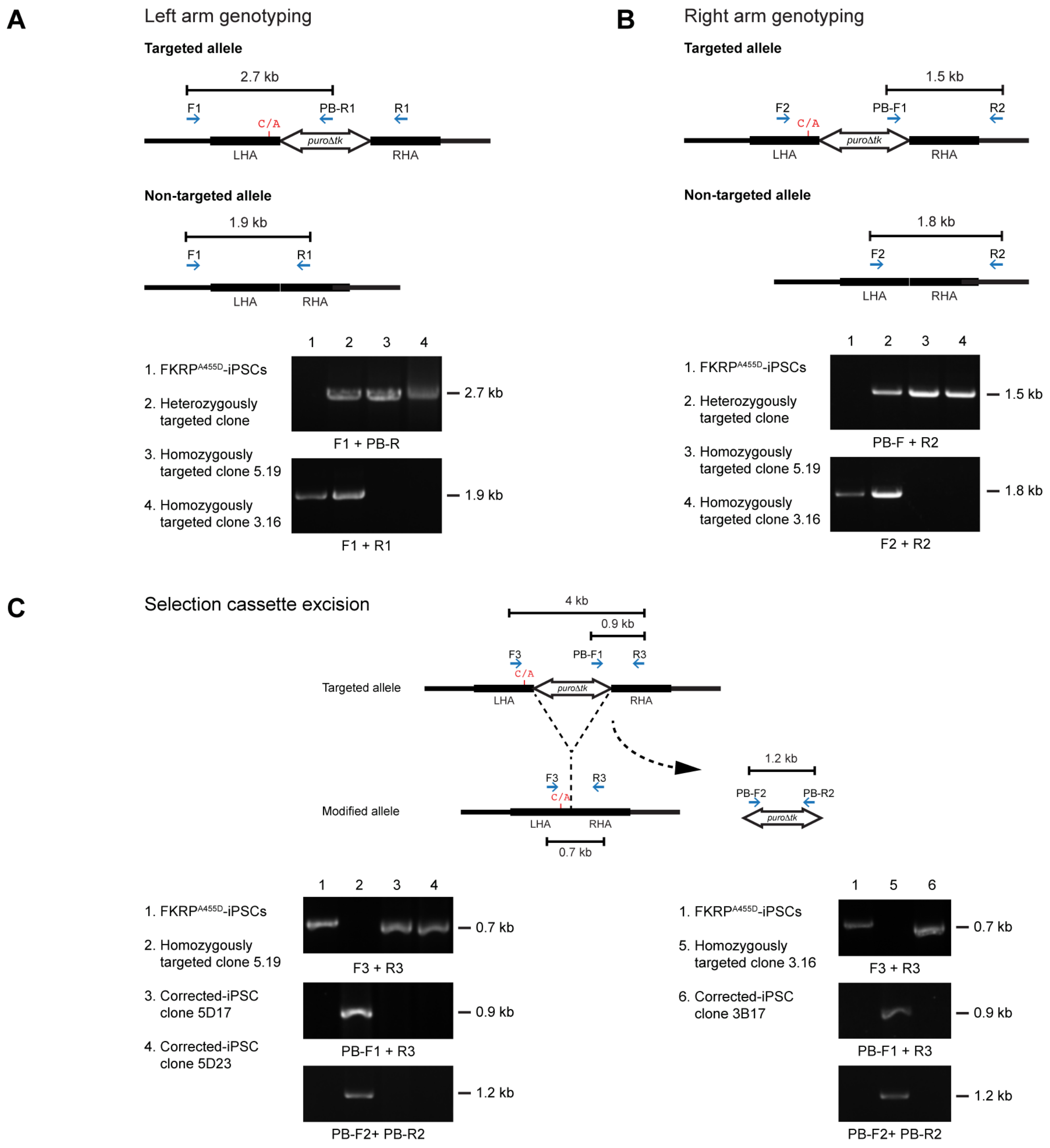

Appendix Figure S2. PCR genotyping of iPSC clones after positive/negative selection. (A) Schematic of targeted and non-targeted alleles with primers designed to detect integration of left homology arm (LHA) from the donor targeting vector. LHA-PCR genotyping confirmed clones 5.19 and 3.16 are homozygously targeted to correct the *FKRP* mutations. (B) Schematic of targeted and non-targeted allele with primers designed to detect integration of right homology arm (RHA) from the donor targeting vector. RHA-PCR genotyping confirmed clones 5.19 and 3.16 are homozygously targeted to correct the *FKRP* mutations. (C) Schematic of selection cassette excision with primers designed to distinguish targeted allele, modified allele and re-integrated selection cassette. PCR genotyping confirmed clones 5D17 and 5D23 derived from targeted clone 5.19 and clone 3B17 derived from targeted clone 3.16 have the selection cassette completely excised without re-integration.

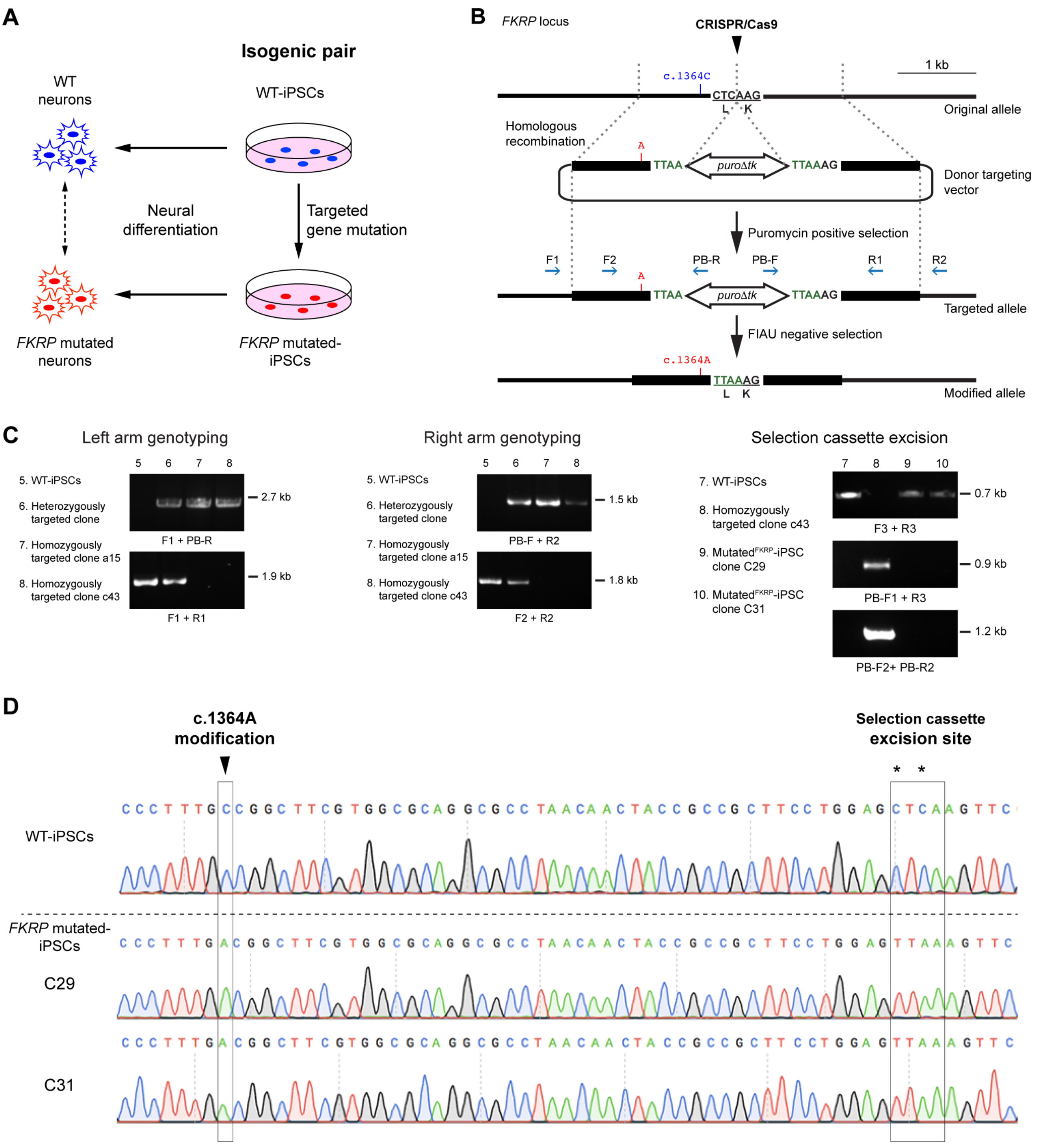

Appendix Figure S3. Knocking in FKRP c.1364C>A (p.A455D) mutation by CRISPR/Cas9 in WT-iPSCs to generate isogenic FKRP mutated-iPSCs. (A) Schematic of targeted gene mutation of *FKRP* in WT-iPSCs and neural differentiation for functional characterization. (B) Genome editing strategy based on CRISPR/Cas9-stimulated homologous recombination, followed by positive (puromycin) and negative (FIAU) selections. LHA and RHA on the targeting donor vector are indicated as black boxes, flanking the *piggyBac* (*PGK-puroΔtk*) selection cassette under the control of *PGK* promoter. PCR genotyping primers are shown as blue arrows. Note that the TTAA sequences are designed to

accommodation the selection cassette excision sites, yet code the same amino acids. (C) See also schematic in Supplementary Figure 2. LHA-PCR genotyping confirmed clones a15 and c43 are homozygously targeted to knock-in the *FKRP* mutations. RHA-PCR genotyping confirmed clones a15 and c43 are homozygously targeted to knock-in the *FKRP* mutations. PCR genotyping confirmed clones C29 and C31 derived from targeted clone c43 have the selection cassette completely excised without re-integration. (D) Sequence analysis shows precise biallelic knock-in of *FKRP* c.1364C>A mutations in two independent iPSC clones (C29 and C31), compared with their parental WT-iPSCs. Selection cassette excision sites are identified in the *FKRP* mutated-iPSC lines.

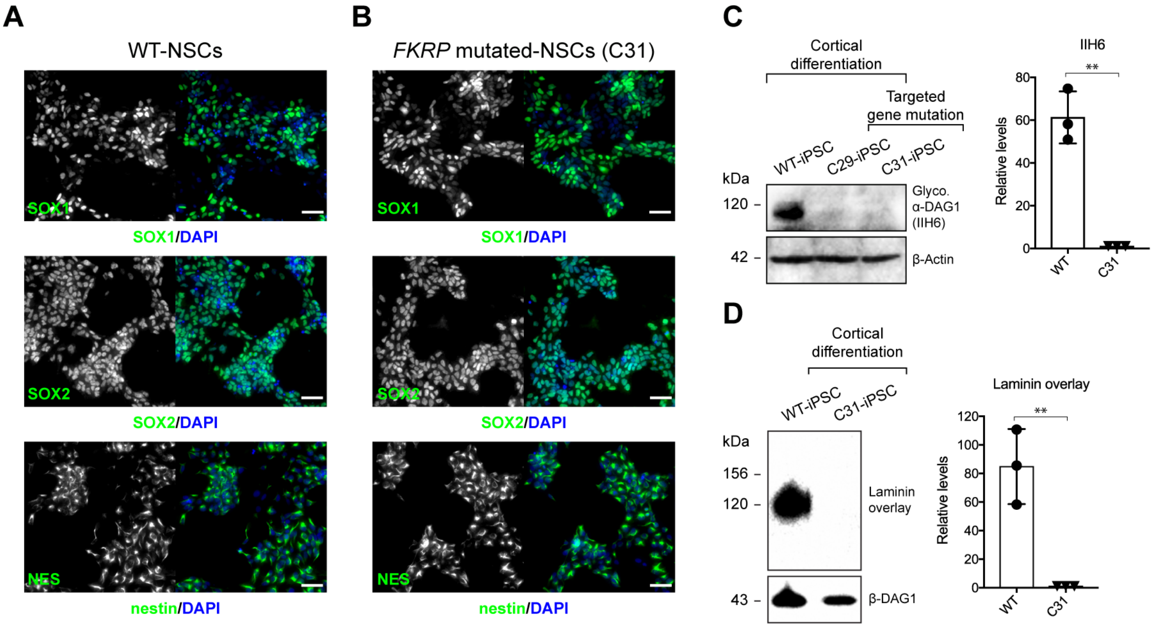

Appendix Figure S4. Characterization of the isogenic pair of WT- and FKRP mutated-iPSCs. (A, B) Representative images of NSCs derived from WT- and FKRP mutated-iPSC lines expressing SOX1, SOX2 and nestin. Scale bars, 50 μm. (C) Representative immunoblots show that targeted gene mutation of FKRP disrupts IIH6 reactivity in iPSC-derived cortical neurons. Intensities of IIH6 reactivity are normalized to β-Actin, indicating mean ± s.d. (n = 3, t-test, ** p<0.01) (D) A representative laminin overlay show that targeted gene mutation abolishes laminin-binding activity in iPSC-derived cortical neurons. Intensities of laminin-binding activity are normalized to β-dystroglycan, indicating mean ± s.d. (n = 3, t-test, * p<0.05, ** p<0.01).

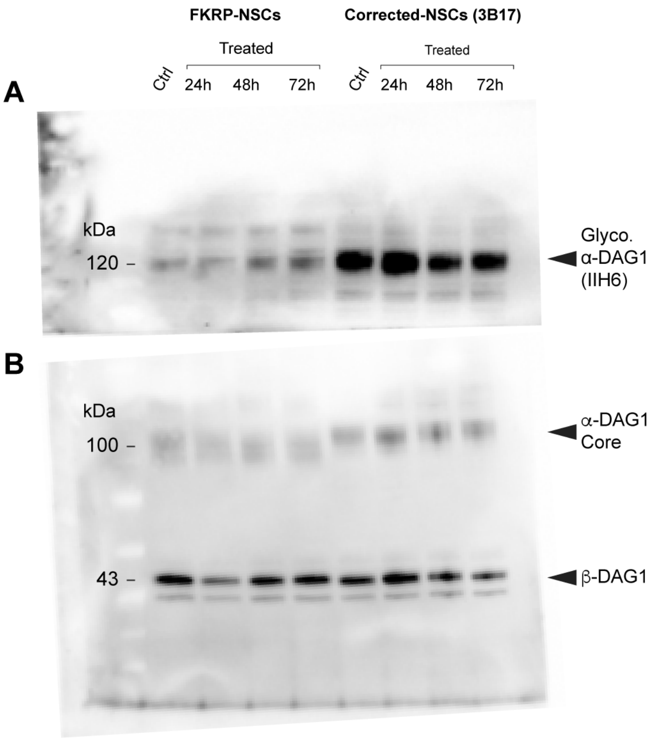

Appendix Figure S5. Uncropped images of representative immunoblots. (A) Immunoblotting showed that FKRP-NSCs treated with 4BPPNit for 48 or 72 hours showed increased IIH6 reactivity, compared with untreated control cells or treated for 24 hours. Note that the augmented IIH6 reactivity by 4BPPNit (pharmacological correction) is not as strong as the IIH6 reactivity in CRISPR-corrected NSCs (genetic correction). Under the same exposure time, the signal intensity of IIH6 reactivity is saturated in corrected-NSCs. (B) Immunoblotting with the polyclonal dystroglycan antibody (AF6868). The molecular weight of dystroglycan core protein in FKRP-NSCs is less than that in corrected-NSCs. The mild increase of IIH6 reactivity in FKRP-NSCs (treated with 4BPPNit for 48 or 72 hours) is not sufficient to shift the core α-DAG1 molecular weight to the same level as that in CRISPR-corrected NSCs.

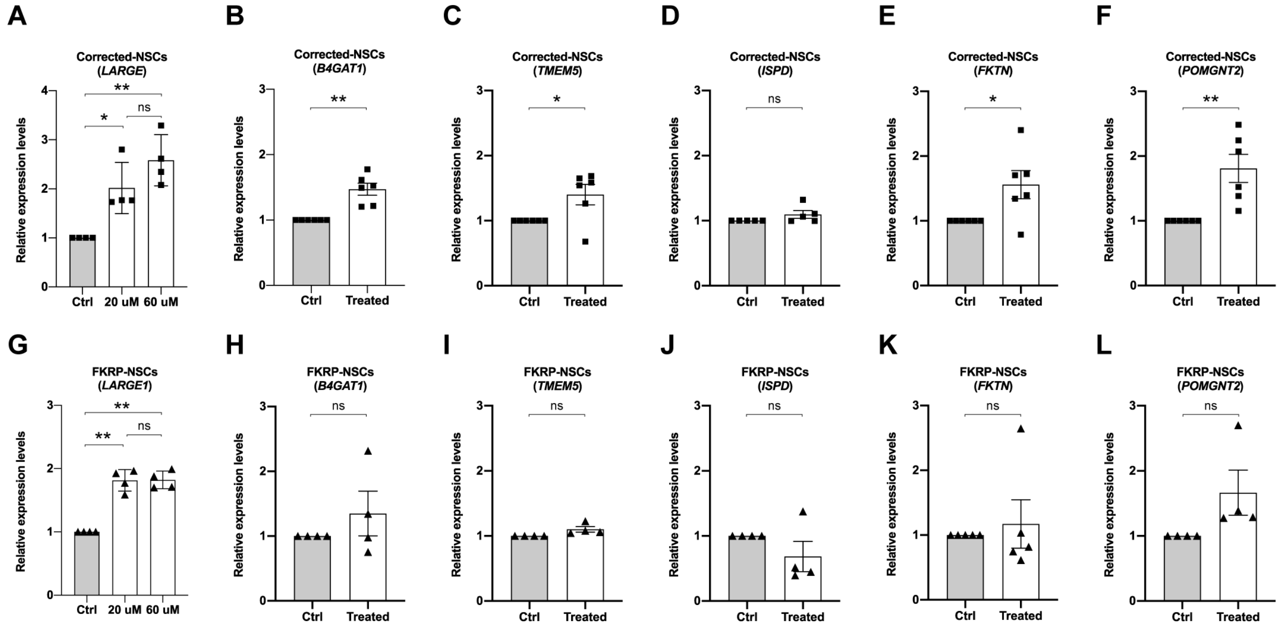

Appendix Figure S6. Gene expression analysis in FKRP- and corrected-NSCs treated with 4BPPNit. (A, G) *LARGE1* expression is significantly upregulated ~2 fold in FKRP- and corrected-NSCs treated with either 20 or 60 μM 4BPPNit for 72 hours, compared with DMSO only controls. No significant difference between 20 and 60 μM 4BPPNit-treated NSCs. (B-F) Except *ISPD* expression, *B4GAT1*, *TMEM5*, *FKTN* and *POMGNT2* expression is significantly upregulated ~1.5 fold in corrected-NSCs treated with 4BPPNit. (H-L) No significant differences in *B4GAT1*, *TMEM5*, *ISPD*, *FKTN, and POMGNT1* expression in FKRP-NSCs treated with 4BPPNit. Unless otherwise stated, NSCs were treated with 20 μM 4BPPNit for 72 hours and compared with DMSO only controls. Error bars indicate mean ± s.e.m (n= 4 - 6). (A, G) One-way ANOVA; (B-F) and (H-L) t-test; ns, not significant; * p<0.05; ** p<0.01.

Appendix Table S1. *FKRP* on-target locus and top 5 predicted off-target sites.

Guide id	On-target	Chr	Strand	Position	Sequence	Mis-matches	Score	Gene
16446360	True	chr19	1	47260113	GGAGCTCAAGTTCGGGCCCGGGG	0	100	NM_024301
16446360	False	chr1	-1	234746867	CGAGTCCAAGTTCGGGCCCGGGG	3	1.502614379	None
16446360	False	chr5	-1	6632784	GGAGCTCAAGATCGTGCCCGAGG	2	0.894322785	NM_001193455
16446360	False	chr10	-1	102983078	AAATCCCAAGTTCGGGCCCGAGG	4	0.813325472	None
16446360	False	chr20	-1	32399512	GAAGGTGAAGTCCGGGCCCGGGG	4	0.488623776	NM_176812
16446360	False	chr5	1	96144043	GGTGCTCAAGTTGGGGACCGAAG	3	0.462897567	None

Appendix Table S2. Targeted gene correction by CRISPR/Cas9.

a) Genotyping of targeted clones

	Clones genotyped	Heterozygously targeted clones	Homozygously targeted clones
Exp. 1	39	14	0
Exp. 2	38	13	3
Total	77	27	3

b) Selection cassette excision

	Clones genotyped	PB excised from both alleles	PB excised without re-integration
Exp. A	18	9	2
Exp. B	16	8	6
Exp. C	24	6	3
Total	58	23	11

Appendix Table S3. Targeted gene mutation by CRISPR/Cas9.

a) Genotyping of targeted clones

	Clones genotyped	Heterozygously targeted clones	Homozygously targeted clones
Exp. 1	10	4	1
Exp. 2	12	6	1
Total	22	10	2

b) Selection cassette excision

	Clones genotyped	PB excised from both alleles	PB excised without re-integration
Exp. A	5	1	0
Exp. B	27	12	2
Total	32	13	2

Appendix Table S4. Primers used for constructing targeting donor vector by Gibson Assembly and generating DNA fragments containing sgRNA target sequence.

**Name**	**Sequence (5' to 3')**
hFKRP_GA_P2rMut (1)	GACTATCTTTCTAGGGTTAACTCCAGGAAGCGGCGGTAGTTGT<br>TAGGCGCCTGCGCCACGAAGCCGGCAAAGGGCAG
hFKRP_GA_P2r_A455D (2)	GACTATCTTTCTAGGGTTAAcTCCAGGAAGCGGCGGTAGTTGT<br>TAGGCGCCTGCGCCACGAAGCCGTCAAAGGGCAG
hFKRP_GA_P1f	CGCGCCGGTACCTTAATTAACACCTGGCCTGCTGGAGCGC
hFKRP_GA_P1r	GCGCTCCAGCAGGCCAGGTGTTAATTAAGGTACCGGCGCG
hFKRP_GA_P2f	ACTACCGCCGCTTCCTGGAGTTAACCCTAGAAAGATAGTC
hFKRP_GA_P2r	GACTATCTTTCTAGGGTTAACTCCAGGAAGCGGCGGTAGT
hFKRP_GA_P3f	GATTATCTTTCTAGGGTTAAAGTTCGGGCCCGGGGTCATC
hFKRP_GA_P3r	GATGACCCCGGGCCCGAACTTTAACCCTAGAAAGATAATC
hFKRP_GA_P4f	GAGGATCGCTTGAGCCCAGGTCTAGAACTAGTGGATCCCC
hFKRP_GA_P4r	GGGGATCCACTAGTTCTAGACCTGGGCTCAAGCGATCCTC
hFKRP_sgRNA_F (3)	ACCGGAGCTCAAGTTCGGGCCCG
hFKRP_sgRNA_R	AAACCGGGCCCGAACTTGAGCTC

Note:

(1) The *FKRP* c.1364C>A mutation is corrected using primer hFKRP_GA_P2rMut, in which the corrected based is underlined.

(2) The *FKRP* c.1364C>A mutation is knocked in using primer hFKRP_GA_P2r_A455D, in which the mutated based is underlined

(3) The sgRNA target sequence for the *FKRP* locus is underlined.

Appendix Table S5. Primers used for PCR genotyping.

**Name**	**Sequence (5' to 3')**
PB-F1	TGGGAAGACAATAGCAGGCA
FKRP-R2	CATGAGTGGAGCTGCGATT
FKRP-F2	AGCTGCTGGACTTGACCTTC
PB-R1	ATAAACCCGCAGTAGCGTGG
FKRP-F1	ATCCCCAACGTGCGTCTG
FKRP-R1	GACTTCTCTCCAAGCCCCTG
FKRP-F3	TGCAGTACAGCGAAAGCAAC
FKRP-R3	GCGATAGAGCCAGGATTTGA
PB-F2	ATGGGGACCGAGTACAAGCC
PB-R2	TATTGGCAAGCAGCCCGTAA

Appendix Table S6. Primers used for qPCR.

**Name**	**Sequence (5' to 3')**
bActin_q1F	GCGAGAAGATGACCCAGATC
bActin_q1R	CCAGTGGTACGGCCAGAGG
NANOG_q1F	TCTGCTTATTCAGGACAGCCC
NANOG_q1R	TGCTGGAGGCTGAGGTATTTC
Oct4-endo_F	CACTGTACTCCTCGGTCCCTTTC
Oct4-endo_R	CAACCAGTTGCCCCAAACTC
REX1_q1F	ACCAGCACACTAGGCAAACC
REX1_q1R	TTCTGTTCACACAGGCTCCA
DAG1_q1F	TCGAGTGACCATTCCAACAGA
DAG1_q1R	GCGCTCACTGAAATGTAATGC
FKRP_q2F	GCCATTGCTCCAAGATGGC
FKRP_q2R	GTTGTAGGCTGGGGGAGTTC
LARGE1_q1F	AGCTTGTCCTGACCAAGACT
LARGE1_q1R	GGTTTCCAAGGTACCAGTCAC
B4GAT1_q1F	CAACTATTCCCGCTGGGTCA
B4GAT1_q1R	CAAAATCAAACCCCGCCACA
TMEM5_q1F	TTGATCCCAGCGATGTGACT
TMEM5_q1R	CACTGGGTGGCATAAAAGATCT

ISPD_q1F	ACCTCCCAGTCAGAAAATGGA
ISPD_q1R	AGCTGACCAATGAGTCCAGA
FKTN_q1F	CACAGTGGCGTGCAGTTAAA
FKTN_q1R	ACTTGCATTGTGAAGTAGAGCC
POMGNT2_q1F	TCACCAGTTCTCAGGATGCC
POMGNT2_q1R	CACATGCTTCCACAGGACC
